# Can Vitamin D supplementation enhance the effectiveness of exercise-induced weight loss in overweight or obese adults? Evidence from integrated transcriptomic and meta-analysis

**DOI:** 10.3389/fnut.2025.1664960

**Published:** 2025-09-30

**Authors:** Tianhang Peng, Zike Zhang, Wanyuan Liang, Jiayi Zhang

**Affiliations:** ^1^Exercise Science School, Beijing Sport University, Beijing, China; ^2^College of Physical Education, Hunan Normal University, Changsha, China; ^3^School of Physical Education, University of Science and Technology Beijing, Beijing, China

**Keywords:** obesity, vitamin D, exercise intervention, transcriptomics, meta-analysis

## Abstract

**Objective:**

Obesity is a major global public health challenge, and Vitamin D deficiency is prevalent among obese individuals. This study aimed to evaluate whether Vitamin D supplementation enhances the effectiveness of exercise-induced weight loss in overweight or obese adults by integrating transcriptomic analysis and meta-analysis.

**Methods:**

Transcriptomic data from the GEO and GTEx databases were integrated for differential gene expression analysis, Gene Ontology (GO)/Kyoto Encyclopedia of Genes and Genomes (KEGG) enrichment, and Gene Set Enrichment Analysis (GSEA). Currently, clinical transcriptomic data regarding the effect of Vitamin D on exercise intervention outcomes in overweight/obese adults is limited. To address this gap, this study utilized cold-induced skeletal muscle shivering as a surrogate model to explore its potential molecular mechanisms. A meta-analysis of eight randomized controlled trials (RCT) involving 481 participants, was conducted to assess the combined effects of exercise and Vitamin D supplementation on body composition and metabolic parameters, with subgroup analyses by age and exercise type.

**Results:**

Transcriptomic analysis revealed abnormal expression of Vitamin D metabolism-related genes in skeletal muscle of obese individuals, with enrichment in pathways such as lipid digestion and absorption. Post-intervention, Vitamin D response pathways were significantly upregulated. The meta-analysis showed that combined intervention had a significant effect on waist circumference (mean difference [MD] = −1.48, 95% CI: −2.02 to −0.94, *p* < 0.05). Subgroup analysis indicated that improvements in body weight and Body Mass Index (BMI) were more pronounced among older adults and those undergoing aerobic exercise.

**Conclusion:**

This study, through integrated high-throughput transcriptomic analysis and meta-analysis, systematically demonstrates that Vitamin D supplementation may enhance skeletal muscle metabolic responsiveness to exercise in overweight or obese adults. The effect appears especially significant in older populations and within aerobic exercise contexts. These findings suggest that Vitamin D supplementation could serve as a synergistic strategy in exercise-based weight loss programs for targeted populations. Future research should focus on individual Vitamin D status, optimization of exercise modalities, and validation of underlying mechanisms to support personalized and precise interventions.

**Systematic review registration:**

https://www.crd.york.ac.uk/PROSPERO/.

## 1 Introduction

Vitamin D is a fat-soluble vitamin that plays a vital role in maintaining bone health and regulating calcium-phosphorus metabolism. It also participates in numerous physiological functions such as immune modulation, anti-inflammatory activity, and antioxidant defense. In recent years, accumulating evidence has shown a close association between Vitamin D deficiency and obesity. Obesity is frequently associated with deficiencies in various micronutrients, with Vitamin D deficiency being the most prevalent (1), which may be attributed to its fat-soluble nature leading to sequestration in adipose tissue, chronic systemic inflammation, and reduced outdoor activity. Therefore, the high global prevalence of Vitamin D deficiency may be one of the key contributors to the rising obesity rates worldwide (2).

Vitamin D deficiency has been linked to multiple metabolic disorders, including dyslipidemia, insulin resistance, and type 2 diabetes (3, 4), all of which are common complications of obesity. Systematic reviews and meta-analyses have suggested that cholecalciferol supplementation may reduce body mass index (BMI) and waist circumference in overweight and obese individuals to a certain extent. However, data on more specific body composition metrics such as body fat percentage and waist-to-hip ratio remain limited (5).

In recent years, researchers have increasingly focused on the non-skeletal effects of Vitamin D, particularly its potential benefits on muscle function. Vitamin D deficiency is associated with muscle weakness, reduced exercise performance, and increased risk of falls, whereas supplementation may enhance muscle strength, improve repair processes, and boost endurance and overall physical function (6). Consequently, it has been proposed that Vitamin D supplementation may potentiate the effects of exercise interventions in improving weight and metabolic outcomes among overweight or obese adults, especially by synergistically optimizing body composition. At the mechanistic level, transcriptomic technology offers new insights into the potential synergy between Vitamin D and exercise. By analyzing gene expression changes under different conditions, transcriptomics can reveal how Vitamin D and exercise jointly regulate pathways in skeletal muscle, adipose tissue, and inflammation. Previous studies have suggested that Vitamin D may modulate signaling pathways such as AMPK, PPARγ, and NF-κB, thereby influencing lipid metabolism, energy homeostasis, and immune function (7–11). Thus, transcriptomics not only facilitates mechanistic understanding but may also serve as a novel molecular biomarker tool for evaluating intervention responses. Although existing studies provide preliminary evidence for the potential value of Vitamin D in obesity management, numerous unresolved issues remain, including optimal dosing, intervention duration, and the ideal combination model with exercise. Therefore, the current study systematically integrates transcriptomic data and clinical trial evidence to evaluate whether Vitamin D supplementation enhances the weight loss effects of exercise in overweight or obese adults.

Compared to previous studies, this review provides a more comprehensive evaluation of the role of Vitamin D supplementation in exercise-induced weight loss in overweight and obese populations. Given the heterogeneity in participant age, exercise modalities, and Vitamin D dosing across studies, we incorporated both transcriptomic and meta-analytic approaches to enhance scientific rigor. This review primarily focuses on the effects of Vitamin D supplementation on body composition and metabolic health in overweight/obese individuals, and further compares its effects on plasma 25-hydroxyvitamin D [25(OH)D] levels when combined with or without exercise. By integrating high-throughput molecular data with clinical evidence, we aim to provide a theoretical and practical framework for developing personalized obesity interventions based on a synergistic “nutrition–exercise–molecular target” strategy, especially for individuals seeking to improve their health through scientifically grounded methods.

## 2 Methods

### 2.1 Transcriptomic data acquisition and analysis

The transcriptomic RNA-seq data of skeletal muscle from 30 obese adults were downloaded from the GEO database [GSE271452, 15 pre-intervention and 15 post-intervention samples, BGISEQ-500 sequencing platform, data type: TPM (Transcripts Per Million)] (12). Additionally, RNA-seq data of 396 normal adult skeletal muscle samples were obtained from the GTEx database^1^ (Illumina HiSeq 2000/2500 sequencing platform, data type: TPM).

RNA-Seq data were subjected to normalization, including the removal of low-expression genes with an average expression below 1 across all samples. The Wilcoxon test was applied to compare gene expression differences between the control and experimental groups. Log2 fold change (logFC) and median differences were calculated, and *p*-values were adjusted using the False Discovery Rate (FDR) method, selecting genes with |logFC| > 1 and FDR < 0.05. These steps ensured data standardization and improved the reliability and accuracy of differential analysis results. ComBat was used to adjust the TPM data from both groups for batch effects, followed by Principal Component Analysis (PCA) to validate the effectiveness of batch effect correction. Subsequently, differentially expressed genes, metabolite abundance, and Kyoto Encyclopedia of Genes and Genomes (KEGG) enrichment analysis were conducted to extract key data. DEGs were identified based on a fold change greater than 0.5 or less than −0.5, with an adjusted *p*-value < 0.1 (13). Differential expression analysis was conducted using R software (version 4.3.3), and *p*-values were adjusted for multiple comparisons using the q-value method.

### 2.2 Registration and public involvement

This systematic review has been registered in the International Prospective Register of Systematic Reviews (PROSPERO) under the registration number CRD42024589772 (14). The literature search followed the PRISMA (Preferred Reporting Items for Systematic Reviews and Meta-Analyses) guidelines (15). As a secondary analysis of published data, this study did not involve direct patient or public participation.

### 2.3 Search strategy

A comprehensive search was conducted in the PubMed, Web of Science, Cochrane Library, EMBASE, and Scopus databases for RCT published up to November 2024. The reference lists of the screened articles were examined to identify additional relevant studies. Search terms included both controlled vocabulary and free-text keywords related to adults, overweight/obesity, vitamin D, and exercise (e.g., exercise, physical training, physical activity, fitness training, aerobic training, or resistance training), and were adapted for each individual database. Specific keywords used for the search are provided in Supplementary Table S1, and the complete electronic search strategies for all databases are detailed in Supplementary Table S2.

### 2.4 Study selection: inclusion and exclusion criteria

The study participants included adults who were overweight (BMI ≥ 25 kg/m^2^) or obese (BMI ≥ 30 kg/m^2^), aged over 18 years (with no upper age limit). These individuals underwent physical activity interventions and took Vitamin D supplements. Participants with obesity-related comorbidities such as type 2 diabetes, hypertension, dyslipidemia, metabolic syndrome, liver diseases (e.g., NAFLD/NASH), and osteoarthritis were not excluded. The interventions included exercise training programs or other measures designed to promote physical activity, in combination with Vitamin D supplementation. The exercise training could be aerobic, resistance, mixed, or high-intensity interval training, in any combination of these types. Exercise training could be supervised, partially supervised, or unsupervised. Vitamin D supplementation was administered in either liquid and/or solid forms. The control groups included those who received only exercise interventions, placebo (e.g., stretching), or Vitamin D supplementation alone. The primary outcomes assessed included changes in weight, BMI, body fat percentage, blood glucose control, and lipid metabolism markers (e.g., triglycerides (TG), High-Density Lipoprotein (HDL), Low-Density Lipoprotein (LDL)), among other metabolic health indicators. Only RCT were included, with the following types of studies excluded: (a) trials conducted in pregnant women; (b) studies focusing solely on dietary patterns, single food interventions, or exercise-only interventions; (c) studies centered on primary prevention of weight gain/obesity; (d) exercise interventions combined with other treatments (e.g., medications).

### 2.5 Data extraction

Data were independently extracted by two reviewers using a standardized form, including study characteristics, participant demographics, intervention details (such as Vitamin D dosage, regimen, and duration), and supervision information (Table 1). Outcome measures included body composition parameters (body weight, BMI, body fat percentage, waist circumference) and metabolic markers (TG, fasting glucose, HDL, LDL, and 25(OH)D). In the event of discrepancies between the two reviewers during data extraction, the differences will first be discussed, and the original literature will be reviewed or consulted with field experts for clarification. If consensus cannot be reached, a third researcher with relevant expertise will be invited to adjudicate. For cases of missing or unclear data, the research team will proactively contact the original authors to obtain the missing information, ensuring the completeness and accuracy of the data.

**Table 1:** Basic characteristics of included studies.

First Author (Year)	Country	Age (years), mean ± SD	Sample size (Female)	BMI (kg/m^2^), Mean ± SD	Intervention characteristics	Duration (weeks)	Frequency (sessions/week)	Vitamin D dose	Outcome measures	Supervision
2022 Fairfield (22)	America	53 ± 1	43(43)	26 ± 1	Resistance training + HMB + Vitamin D3	12	3	2000 IU /d	①②③	Supervised
2022 Nazarabadi (23)	Iran	53 ± 5	46(46)	34.4 ± 1	Aerobic training + Vitamin D	8	3	50,000 IU /w	④⑤⑥	Not reported
2019 Bahador (24)	Iran	28.1 ± 2.7	40(40)	30 ± 0.8	TRX training + Calcium + Vitamin D	8	3	1,000 IU /w	③⑤⑦⑧	Supervised
2020 Kallantar (25)	Iran	28.4 ± 3	40 (40)	27.1 ± 1.4	Resistance training + Vitamin D	8	3	1,000 IU /w	①②⑨	Unsupervised
2021 Heba (26)	Egypt	35.1 ± 2.5	45 (45)	34.2 ± 1.5	Intermittent aerobic exercise + Vitamin D	12	3	50,000 IU /w	②⑨	Supervised
2014 Amely (27)	Netherlands	63. 6 ± 5.6	94 (66)	33.6 ± 4.4	Resistance training + Vitamin D supplement	13	3	Unclear	①②③④	Supervised
2021 Robert (28)	Netherlands	66.3 ± 6.2	123 (47)	33.1 ± 4.5	Vitamin D-fortified protein + lifestyle intervention	13	3	Unclear	①②④⑥	Supervised
2023 Jakub (29)	Australia	60 ± 6	50 (19)	30.6 ± 5.7	Vitamin D + Multimodal exercise	12	3	4,000 IU /d	①②③④⑤⑥⑦⑧	Supervised

Primary outcome indicators:① Body weight;② Body mass index (BMI);③ Body fat percentage;④ Waist circumference;⑤ Triglycerides (TG);⑥ Fasting blood glucose;⑦ High-density lipoprotein (HDL);⑧ Low-density lipoprotein (LDL);⑨ serum 25-hydroxyvitamin D [25(OH)D].

### 2.6 Data synthesis and statistical analysis

Meta-analyses were performed using RevMan 5.4. Weighted mean differences (WMDs) or standardized mean differences (SMDs) were calculated for each outcome. Depending on heterogeneity (I^2^ statistic), a fixed-effect model was used for I^2^ < 50%, and a random-effects model for I^2^ ≥ 50% (16). To further explore the variability in intervention effects, we adopted 60 years as the threshold for the elderly group (<60 years vs. ≥60 years), in accordance with high-quality studies and systematic reviews. This age cutoff aligns with international standards and effectively reflects age-related physiological and cognitive changes (17, 18). Additionally, subgroup analyses were conducted based on exercise types (aerobic, resistance, and multimodal), with statistical tests performed (19). Robustness was evaluated using one-by-one sensitivity analyses. Publication bias was assessed through visual inspection of funnel plots, and Egger’s test was performed if asymmetry was evident and the number of studies exceeded 10.

### 2.7 Risk of bias assessment

Study quality was independently assessed by two reviewers using the Cochrane Risk of Bias Tool (20). The evaluation covered random sequence generation, allocation concealment, blinding, completeness of outcome data, and selective reporting (21). Disagreements were resolved by a third reviewer.

## 3 Results

### 3.1 Transcriptomic analysis

**Figure 1:**
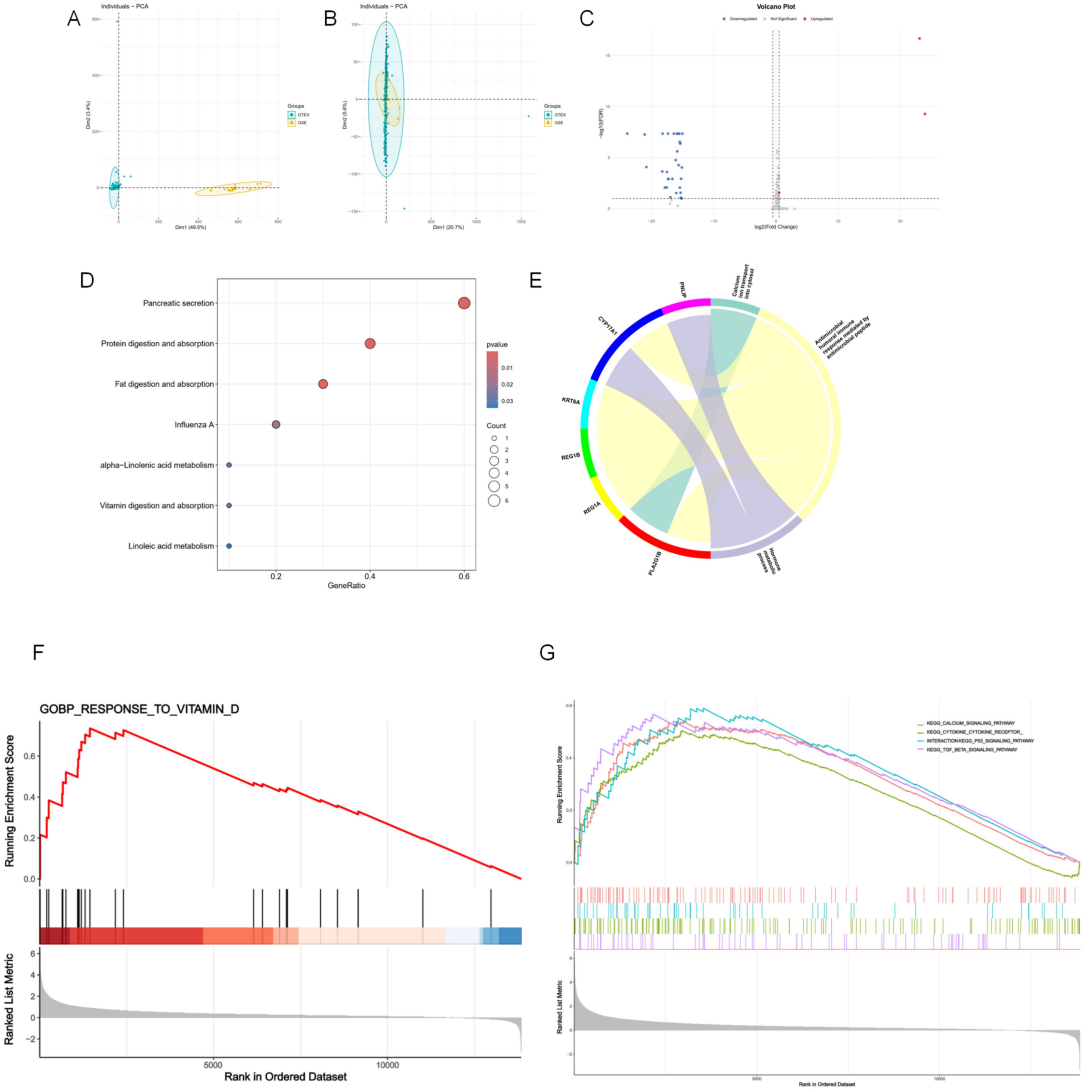
Transcriptomic analysis of GSE271452 and GTEx skeletal muscle data. **(A,B)** Principal component analysis (PCA) of skeletal muscle transcriptomic data from GSE271452 and GTEx before and after batch effect correction. **(C)** Volcano plot of differentially expressed genes in merged data (logFC > 0.5 or < −0.5, FDR < 0.1). **(D)** KEGG pathway enrichment of differentially expressed genes. **(E)** GO enrichment highlighting Vitamin D metabolism-related pathways and associated genes. **(F,G)** GSEA results of skeletal muscle transcriptomes in obese adults before and after cold exposure, showing enrichment of Vitamin D response genes and key signaling pathways.

Due to the lack of healthy control samples in the GSE271452 dataset, transcriptomic data from skeletal muscle tissue in the GTEx database were integrated for batch effect correction and normalization (Figures 1A,B). Differential expression analysis identified 36 genes that were significantly differentially expressed between obese and healthy adult skeletal muscle samples (Figure 1C). KEGG pathway enrichment analysis revealed that these genes were significantly enriched in several metabolism-related pathways (Figure 1D), including fat and protein digestion and absorption, pancreatic secretion, and fatty acid metabolism. Although these pathways were not directly enriched in Vitamin D digestion or absorption, their functional alterations may indirectly impair Vitamin D metabolism and bioavailability. For example, reduced lipid absorption efficiency could decrease the uptake of fat-soluble vitamins such as Vitamin D, and changes in genes related to pancreatic function and protein digestion may affect gut physiology, further exacerbating Vitamin D deficiency. GO (Gene Ontology) functional annotation supported this hypothesis, showing that obese individuals exhibited significant alterations in gene expression related to Vitamin D-associated biological processes, such as calcium ion transport, hormone metabolism, immune response, inflammation regulation, and cellular structural maintenance (Figure 1E). These mechanistic alterations may collectively contribute to impaired Vitamin D metabolism in obese populations, increasing the risk of metabolic syndrome and its related complications. Therefore, Vitamin D supplementation strategies for individuals with obesity should be tailored with consideration of their distinct molecular expression profiles.

Furthermore, clinical transcriptomic data addressing the key question of whether Vitamin D enhances the effects of exercise interventions remain scarce. To explore potential molecular mechanisms, we employed cold-induced skeletal muscle shivering as a surrogate model. Previous studies have shown that cold exposure can stimulate low-intensity muscle contractions, elevate energy expenditure, and improve obesity-related metabolic outcomes. GSEA (Gene Set Enrichment Analysis) was performed on skeletal muscle transcriptomic data from obese adults before and after cold exposure. The results showed significant enrichment of Vitamin D-responsive genes post-intervention, alongside activation of several KEGG pathways, including calcium signaling, cytokine-cytokine receptor interaction, p53 signaling, and TGF-*β* signaling (Figures 1F,G). These findings suggest that cold-induced muscle activity may upregulate Vitamin D response genes and downstream pathways, thereby improving skeletal muscle metabolic function, enhancing lipid metabolism, and promoting tissue repair. This provides an important molecular clue for further investigations into how Vitamin D may synergize with exercise interventions to improve metabolic health in obese individuals.

### 3.2 Meta-analysis results

#### 3.2.1 Literature search

**Figure 2:**
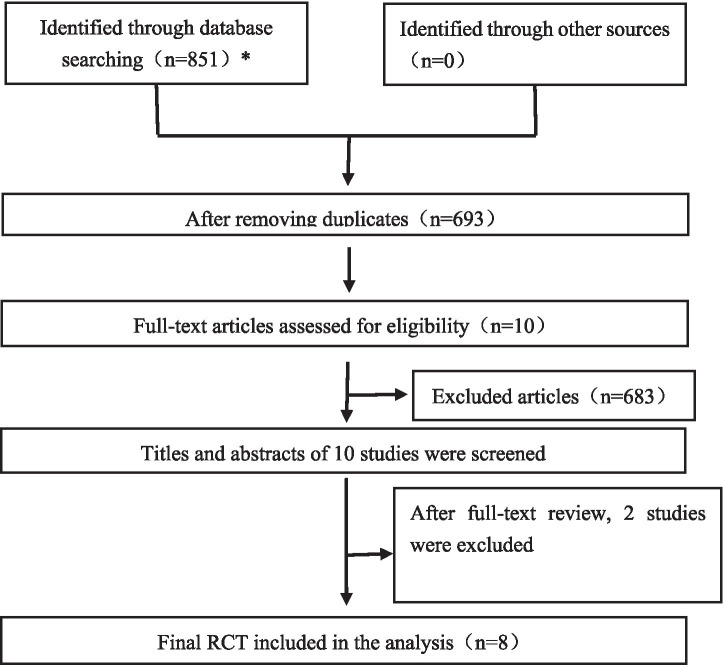
PRISMA flowchart of study selection. The number of studies retrieved from each database was as follows: PubMed (*n* = 38), Web of Science (*n* = 90), Cochrane Library (*n* = 160), Scopus (*n* = 454), and Embase (*n* = 109).

A total of 851 potentially eligible records were identified through systematic database searching. After removing 158 duplicates, full-text screening was conducted for 10 articles to determine eligibility. Of these, 8 studies met the inclusion criteria and were incorporated into both the systematic review and the meta-analysis (22–29). Figure 2 illustrates the flowchart of the study’s search and selection process.

#### 3.2.2 Characteristics and risk of bias of included studies

**Figure 3:**
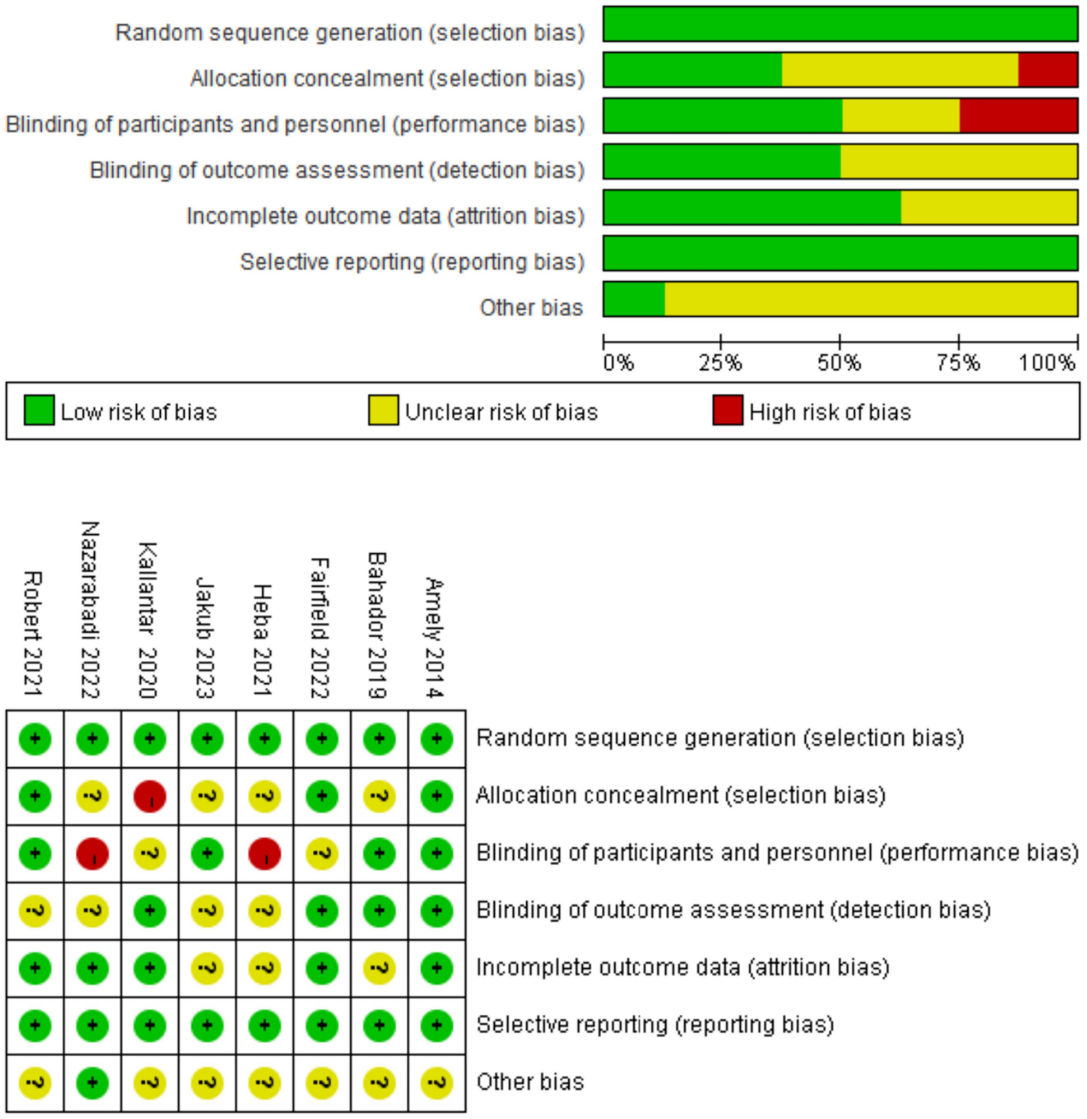
Risk of bias assessment for included studies. All eight included studies were judged to have adequate randomization procedures. Three studies provided details on allocation concealment. Four studies adopted double-blinding, and four described blinded outcome assessments. Most studies reported complete outcome data and showed no evidence of selective reporting.

Table 1 summarizes the key characteristics of the eight included studies, published between 2011 and 2023, encompassing a total of 481 participants. Sample sizes ranged from 40 to 123; the proportion of female participants ranged from 38 to 100%. The average age of participants varied from 28 to 66 years, with baseline BMI ranging from 26.1 to 34.2 kg/m^2^. Exercise interventions lasted between 8 and 13 weeks, conducted three times per week. Vitamin D supplementation dosages varied substantially across studies. Interventions included aerobic, resistance, and multimodal training, most of which were supervised. Additionally, in studies by Fairfield (22) and Heba (26), all participants were Vitamin D deficient at baseline. Detailed risk of bias assessments are illustrated in Figure 3.

#### 3.2.3 Effects of exercise combined with Vitamin D supplementation on body composition in overweight/obese adults

Five studies assessed changes in body weight. The random-effects meta-analysis showed that Vitamin D supplementation did not significantly enhance the overall weight reduction effect of exercise [MD = −1.84, 95% CI (−4.85, 1.18)]. However, subgroup analysis revealed that in participants undergoing aerobic exercise, Vitamin D supplementation significantly enhanced weight loss [MD = −2.15, 95% CI (−2.91, −1.38)]. Similar benefits were observed in individuals aged ≥60 years [MD = −2.09, 95% CI (−2.85, −1.33)]. For BMI, the meta-analysis of six studies also showed no overall significant effect [MD = −0.45, 95% CI (−1.54, 0.65)]. Nevertheless, subgroup analysis indicated that Vitamin D supplementation significantly improved BMI reduction when combined with multimodal exercise [MD = −0.50, 95% CI (−0.73, −0.28)], with a similar effect observed in older adults [MD = −0.50, 95% CI (−0.73, −0.28)]. For body fat percentage, results from four studies indicated no significant additive effect of Vitamin D on the reduction induced by exercise [MD = −0.35, 95% CI (−3.33, 2.64)]. In contrast, for waist circumference, data from four studies using a fixed-effects model showed a significant additional reduction with Vitamin D supplementation [MD = −1.48, 95% CI (−2.02, −0.94)] (see Table 2).

**Table 2:** Meta-analysis results on the effects of exercise combined with Vitamin D supplementation in overweight or obese adults.

Outcome measure	Sample size	Heterogeneity		Effect model	Meta-analysis result	
		*I* ^2^(%)	*p*-value		MD (95%CI)	*p*-value
Body weight	307	71	<0.05	Random	−1.84 (−4.85, 1.18)	0.23
A	173	0	0.85	Fixed	−2.1 (−2.91, −1.38)	<0.05
R	134	0	0.85	Fixed	−1.18 (−7.68, 5.32)	0.72
≥60	267	0	0.39	Fixed	−2.09 (−2.85, −1.33)	<0.05
<60	40	92	<0.05	Random	−2.70 (−11.03, 5.63)	0.52
BMI	337	78	<0.05	Random	−0.45 (−1.54, 0.65)	0.42
M	173	0	0.49	Fixed	−0.50 (−0.73,-0.28)	<0.05
R	134	87	<0.05	Random	−0.35 (−2.50, 1.79)	0.75
≥60	267	0	0.78	Fixed	−0.50 (−0.73, −0.28)	<0.05
<60	70	93	<0.05	Random	−0.31 (−3.54, −2.92)	0.85
Body fat percentage	184	84	<0.05	Random	−0.35 (−3.33, 2.64)	0.82
Waist circumference	290	35	0.2	Fixed	−1.48 (−2.02, −0.94)	<0.05
TG	93	74	<0.05	Random	0.52 (−0.28, 1.41)	0.26
Fasting glucose	196	88	<0.05	Random	−0.53 (−1.51, −0.44)	0.28
HDL	70	0	0.66	Fixed	−6.35 (−13.83, 1.14)	0.1
LDL	70	0	0.48	Fixed	1.89 (−18.13, 21.91)	0.85
25(OH)D	50	79	<0.05	Random	2.36 (−0.61, 5.32)	0.12

A, Aerobic exercise; R, Resistance exercise; M, Multimodal exercise (a combination of different types of exercise such as aerobic exercise, resistance training, and flexibility training within the intervention program); ≥60: Participants aged 60 years and above; <60: Participants aged below 60 years.

#### 3.2.4 Effects of exercise combined with Vitamin D supplementation on metabolic markers

In terms of metabolic parameters, meta-analysis of three studies showed that Vitamin D supplementation did not significantly enhance the effect of exercise in reducing triglyceride (TG) levels [MD = 0.52, 95% CI (−0.28, 1.41)]. Likewise, no significant difference was found for fasting glucose [MD = −0.53, 95% CI (−1.51, 0.44)]. HDL and LDL analyses were based on two studies. The results showed no significant improvement in HDL [MD = −6.35, 95% CI (−13.83, 1.14)] or reduction in LDL [MD = 1.89, 95% CI (−18.13, 21.91)] with combined intervention. Regarding Vitamin D status, two studies assessed 25(OH)D concentrations. Meta-analysis showed that exercise combined with supplementation did not significantly increase 25(OH)D levels compared to supplementation alone [MD = 2.36, 95% CI (−0.61, 5.32)] (Table 2).

#### 3.2.5 Publication bias assessment

Funnel plots were used to assess publication bias across all outcome variables. No evidence of publication bias was observe (Supplementary Figures S1–S9).

## 4 Discussion

Vitamin D is widely used in weight management among obese populations due to its role in chronic disease prevention and metabolic regulation (30). Exercise, as a safe and multifaceted intervention, can improve energy metabolism, mood states, and disease risk (31–33). In recent years, increasing research has attempted to incorporate Vitamin D supplementation into exercise programs to enhance their intervention effects. Transcriptomic analysis provides molecular-level evidence for this hypothesis. By integrating data from GSE271452 and GTEx skeletal muscle samples, 36 differentially expressed genes were identified, and enrichment analysis indicated that Vitamin D metabolic disturbances in obesity are primarily mediated through pathways related to lipid digestion and absorption, pancreatic secretion, calcium ion transport, and hormone metabolism. These disturbances may affect nutrient absorption in the gut and exacerbate Vitamin D deficiency. In selecting the experimental model, this study used cold-induced muscle shivering to simulate exercise intervention. Although this approach offers advantages in terms of ethics and control, its physiological mechanisms differ from those of real exercise interventions. Cold-induced energy metabolism regulation involves two mechanisms: shivering thermogenesis through muscle shivering and non-shivering thermogenesis mediated by brown adipose tissue (34, 35). In contrast, voluntary exercise is a more complex physiological adaptation process, involving active muscle contraction, cardiovascular system responses, and long-term metabolic reprogramming (36). The cold-induced muscle shivering model showed that, after intervention, genes related to Vitamin D response in the skeletal muscle of obese participants were significantly upregulated, activating metabolic pathways such as p53, TGF-*β*, and calcium signaling. This suggests that Vitamin D may be involved in energy metabolism regulation by modulating muscle remodeling and mitochondrial function. These findings are consistent with previous studies on Vitamin D’s role in regulating muscle synthesis, lipid metabolism, and insulin sensitivity (37–43), further supporting its potential mechanistic role in combined interventions. Although this model offers some insights, the indirect connection to actual exercise physiology needs further clarification in future studies, particularly concerning how Vitamin D might regulate muscle remodeling (44–46), mitochondrial function (47–49), and calcium signaling (50, 51) in the context of energy metabolism. Such explanations may help better understand the mechanisms underlying the role of Vitamin D in exercise interventions.

The results of the meta-analysis showed that, although Vitamin D combined with exercise did not demonstrate consistent improvements in weight, BMI, or body fat percentage, it did show certain advantages in reducing waist circumference (30). Subgroup analysis further revealed that the combined intervention was more effective in controlling weight and BMI in older adults (≥60 years) and in participants engaging in aerobic exercise, suggesting that individual characteristics and exercise modalities may modulate intervention effects. Additionally, previous studies have indicated that Vitamin D deficiency is strongly associated with obesity, metabolic syndrome, and cardiovascular disease, particularly in women and individuals with abdominal obesity (2, 52). Although calcitriol supplementation can modestly reduce BMI and waist circumference (53), its direct impact on weight remains limited. Combined interventions may work through multiple mechanisms, such as enhancing Vitamin D receptor (VDR) expression (54), modulating leptin levels (55), and improving gut microbiota and bile acid metabolism (40), thereby synergistically promoting fat metabolism. However, the results on whether exercise significantly elevates serum 25(OH)D levels remain inconsistent. Some studies suggest that endurance or mixed training may be effective, while resistance training alone has limited effects (56–59). Given that Vitamin D in obese populations is primarily stored in adipose tissue with limited bioavailability (60), combined interventions may be of greater clinical significance for this group (22, 26, 61). At the same time, Vitamin D levels are influenced by various factors such as diet, supplement intake, skin color, and sun exposure (62, 63). Therefore, future research should focus on variables such as VDR expression, baseline Vitamin D status, exercise intensity, and intervention duration, and use standardized, long-term follow-up RCT to further clarify the adaptability and mechanistic basis of “Vitamin D + exercise” interventions in different populations.

Moreover, in this study’s meta-analysis, we observed high I^2^ values for some outcome measures, suggesting significant heterogeneity across studies. This heterogeneity may stem from differences in demographic characteristics such as age, gender distribution, baseline Vitamin D status, and methodological differences such as exercise modalities (e.g., aerobic, resistance, or multimodal training), intervention duration, frequency, and supplementation doses (ranging from 1,000 IU/d to 50,000 IU/w). Higher doses of Vitamin D may have a more significant impact on muscle function and fat metabolism, but this was not consistently reflected in the current analysis (29, 64, 65). To reduce bias due to heterogeneity, we used a random-effects model for the summary estimate and further explored the moderating effects of individual characteristics and intervention modalities through subgroup analysis. Notably, subgroup results showed that Vitamin D combined with exercise had a more significant impact on weight and BMI improvement in older adults (≥60 years) and those participating in aerobic exercise, suggesting that age has a clear physiological boundary, which is often used in previous studies to distinguish populations with significant differences in metabolic sensitivity. However, the studies included in the current analysis used multiple different serum 25(OH)D cutoffs (25 nmol/L, 50 nmol/L, and 75 nmol/L), and some studies cited multiple thresholds within the same paper (29), which limited the feasibility of this subgroup analysis. Although theoretically, both interventions may synergistically affect fat generation, reduce inflammation, and show potential in improving metabolic indicators, high-quality RCT are currently lacking to confirm their advantages in weight or body fat reduction (66–68). As such studies increase, especially with standardized reporting of Vitamin D status, this suggestion will have significant potential to refine target populations and intervention strategies.

In terms of transcriptomic analysis, although batch effects between the GSE and GTEx databases were corrected using the ComBat algorithm, and the comparability of the integrated data was verified through PCA, there remain certain differences in sample size and individual characteristics (such as BMI levels, gender composition, etc.), which may influence the robust identification of differentially expressed genes. Particularly in the exploration of mechanisms using cold-induced muscle shivering as a model for exercise intervention, while this model does not fully replicate the metabolic activation mechanisms of voluntary exercise, it offers a certain degree of substitutability in terms of controllability, ethical applicability, and biological energy mobilization. Therefore, it can serve as an approximate research tool to study the involvement of Vitamin D in skeletal muscle metabolic remodeling. It is also worth noting that some of the included studies did not provide detailed information on Vitamin D formulation, bioavailability, intervention duration, or adherence to supplementation, limiting the in-depth analysis of the dose–response relationship. Furthermore, the lack of baseline 25(OH)D status reports restricts the evaluation of sensitivity in Vitamin D deficiency subgroups. Future research should focus on intervention stratification based on individual nutritional status, particularly in individuals with low or deficient Vitamin D levels, by combining multimodal exercise interventions (i.e., composite regimens involving various types of exercise). This approach could enhance the comparability and external validity of intervention outcomes.

The clinical significance of this study lies in the finding that Vitamin D supplementation can amplify the weight loss effects induced by exercise, particularly in improving waist circumference and promoting reductions in weight and BMI. These benefits are especially pronounced in older adults and overweight or obese individuals undergoing aerobic exercise interventions. Our findings suggest that combining exercise with Vitamin D supplementation may represent an effective strategy for optimizing weight management and metabolic health. Therefore, future studies should further define the optimal exercise protocols, explore the ideal dosage and duration of Vitamin D supplementation, and examine its effects on other metabolic markers, such as blood glucose and lipid profiles, to enhance its clinical efficacy in improving body composition and metabolic health. Additionally, the integration of multi-omics data and nutritional behavior monitoring technologies to systematically elucidate the mechanisms underlying the combined effects of Vitamin D and exercise, as well as individual response differences, will provide stronger empirical support for the development of precise and effective exercise-nutrition combined interventions.

## 5 Conclusion

This study systematically evaluated the effects of Vitamin D supplementation on exercise-based interventions in overweight and obese adults, and explored potential underlying mechanisms using transcriptomic data. Transcriptomic analysis revealed that Vitamin D-related gene expression in skeletal muscle is significantly altered in individuals with obesity, primarily through pathways involving fat absorption, pancreatic function, calcium transport, and hormone metabolism. Additionally, a cold-induced muscle shivering model indicated significant enrichment of Vitamin D-responsive genes and their downstream pathways following intervention, suggesting a potential role in muscle metabolic regulation. Meta-analysis results showed that combining exercise with Vitamin D supplementation significantly improved waist circumference reduction, with more pronounced effects on weight and BMI observed in older adults and those engaging in aerobic training—supporting a possible synergistic effect. However, no significant enhancements were observed in glucose or lipid metabolism, which may be due to individual differences in Vitamin D metabolism, baseline levels, or intervention protocols. In summary, Vitamin D supplementation may enhance exercise-induced benefits by improving skeletal muscle metabolism and adaptive responses, thereby synergistically supporting weight-loss interventions. Future research should focus on personalized Vitamin D status, intervention intensity and duration, and molecular validation, aiming to develop more precise and effective nutrition-exercise strategies for overweight and obese populations.

## Data Availability

Publicly available datasets were analyzed in this study. This data can be found in the NCBI GEO repository: https://www.ncbi.nlm.nih.gov/geo/query/acc.cgi?acc=GSE271452 and the GTEX repository: https://gtexportal.org/home/downloads/adult-gtex/bulk_tissue_expression.

## Footnotes

^1^ https://www.gtexportal.org/home/
