# Supplementary material for: Can Vitamin D supplementation enhance the effectiveness of exercise-induced weight loss in overweight or obese adults? Evidence from integrated transcriptomic and meta-analysis
Source: Front Nutr. 2025 Sep 30;12:1664960. doi: 10.3389/fnut.2025.1664960 (PMC12519668; doi:10.3389/fnut.2025.1664960)
Supplement: Supplementary file 1 [file Supplementary_file_1.docx]

**Supplementary Material**

**Table S1 Medical Subject Headings and Entry Terms**

**
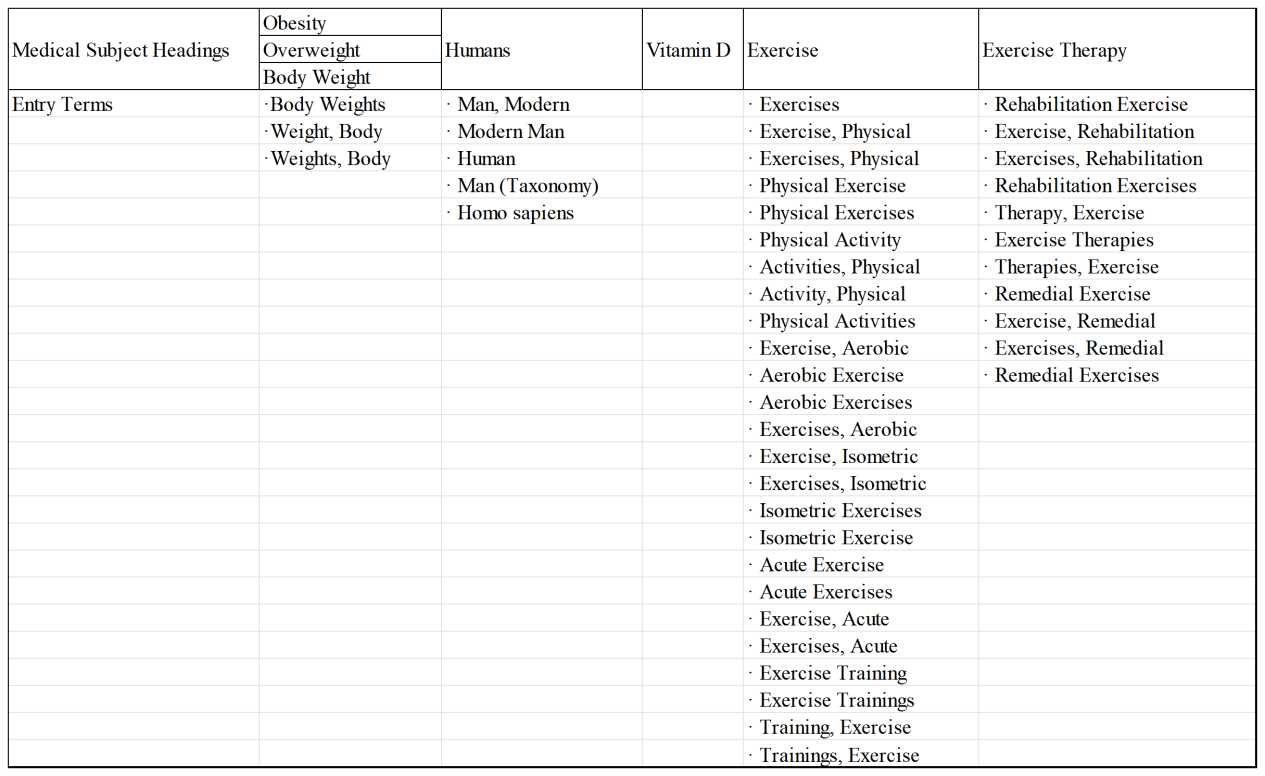
**

**Table S2** **Search strategy used for all searched databases**

Literature search in PubMed, Embase, Web of Science,Scoups, The Cochrane library. Searches performed 2024-11.


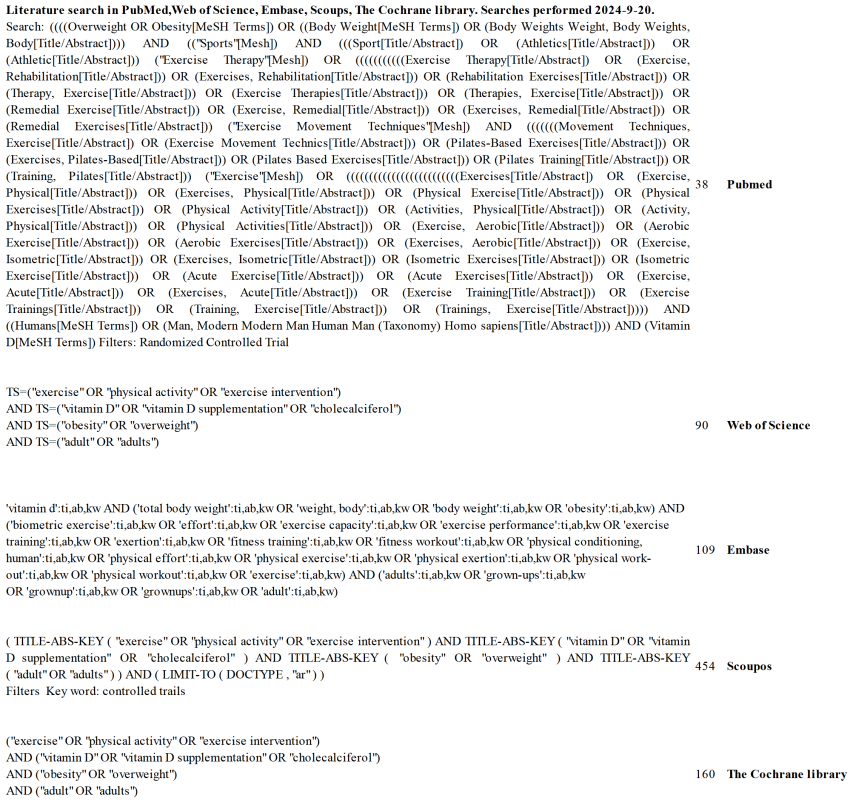

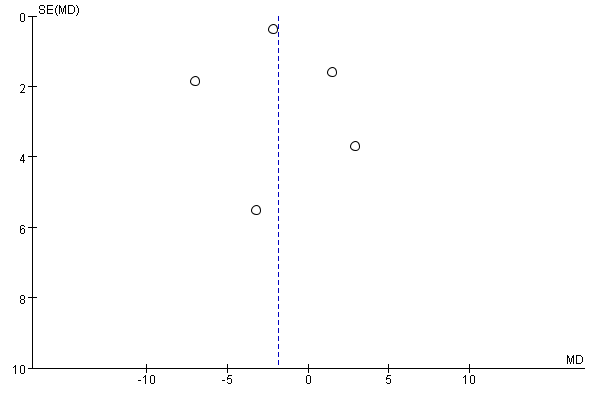


**Figure S1. Funnel plot assessing the effect of exercise and vitamin D supplementation on body weight in overweight/obese adults.**


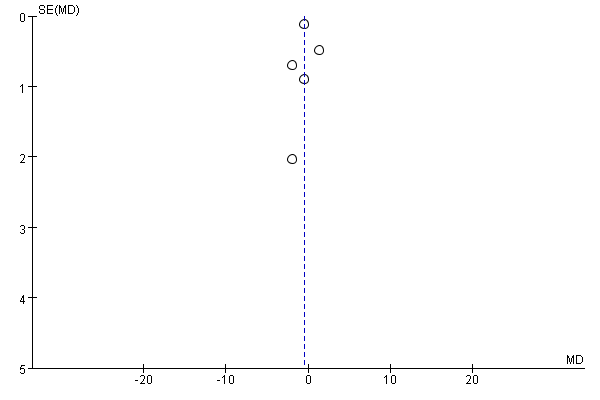


**Figure S2. Funnel plot assessing the effect of exercise and vitamin D supplementation on BMI in overweight/obese adults.**


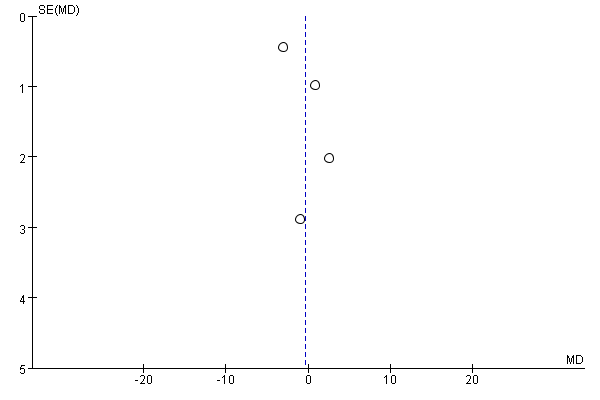


**Figure S3. Funnel plot assessing the effect of exercise and vitamin D supplementation on body fat percentage in overweight/obese adults.**


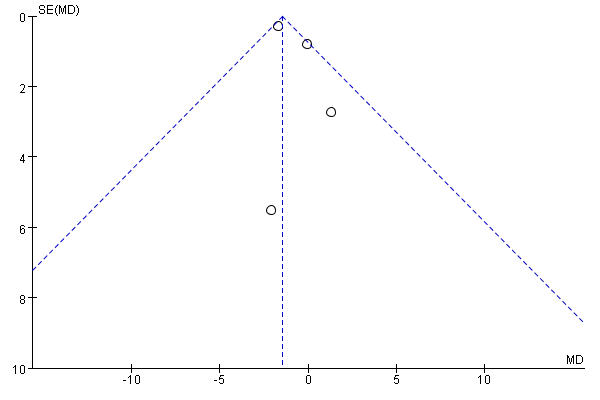


**Figure S4. Funnel plot assessing the effect of exercise and vitamin D supplementation on waist circumference in overweight/obese adults.**


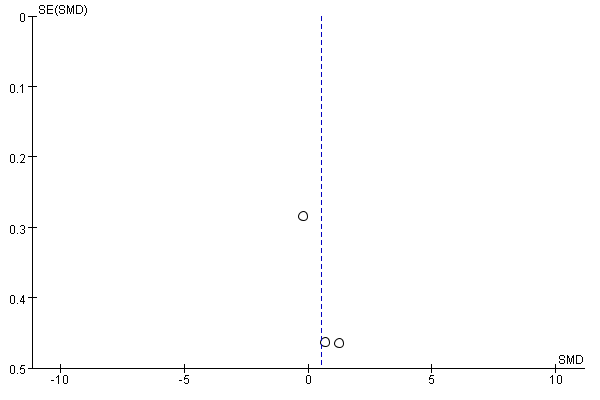


**Figure S5. Funnel plot assessing the effect of exercise and vitamin D supplementation on triglycerides in overweight/obese adults.**


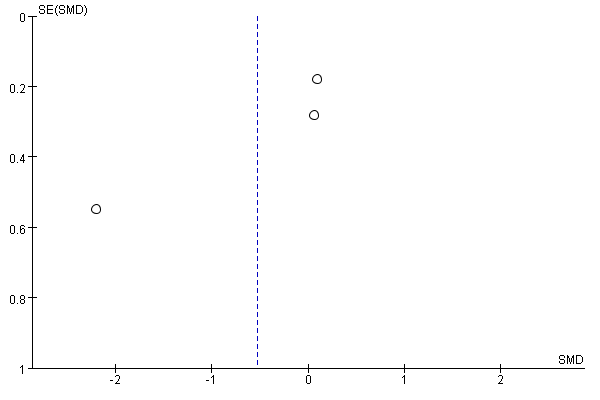


**Figure S6. Funnel plot assessing the effect of exercise and vitamin D supplementation on fasting blood glucose in overweight/obese adults.**


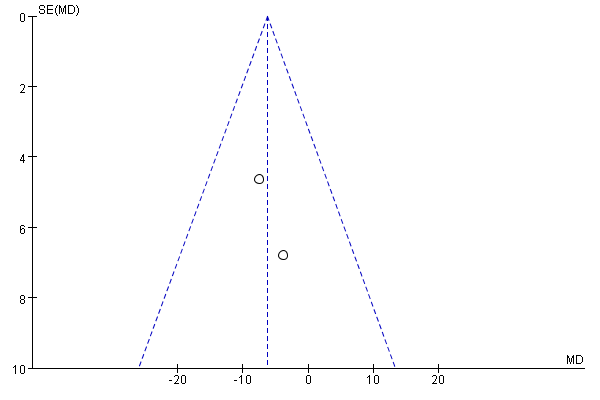


**Figure S7. Funnel plot assessing the effect of exercise and vitamin D supplementation on high-density lipoprotein in overweight/obese adults.**


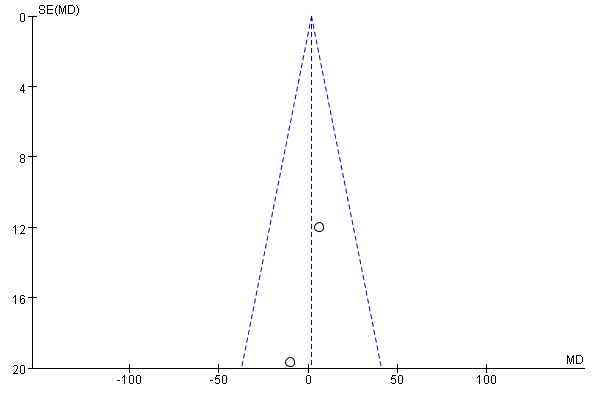
**Figure S8. Funnel plot assessing the effect of exercise and vitamin D supplementation on low-density lipoprotein in overweight/obese adults.**


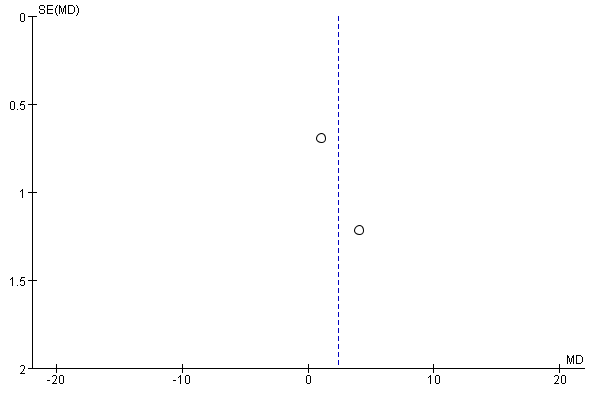


**Figure S9. Funnel plot assessing the effect of exercise and vitamin D supplementation on 25-OH-VitD levels in overweight/obese adults.**
